# Open-access bacterial population genomics: BIGSdb software, the PubMLST.org website and their applications

**DOI:** 10.12688/wellcomeopenres.14826.1

**Published:** 2018-09-24

**Authors:** Keith A. Jolley, James E. Bray, Martin C. J. Maiden

**Affiliations:** 1 Department of Zoology, University of Oxford, Oxford, OX1 3PS, UK

**Keywords:** Database, population annotation, evolution, epidemiology, public health

## Abstract

The PubMLST.org website hosts a collection of open-access, curated databases that integrate population sequence data with provenance and phenotype information for over 100 different microbial species and genera.  Although the PubMLST website was conceived as part of the development of the first multi-locus sequence typing (MLST) scheme in 1998 the software it uses, the Bacterial Isolate Genome Sequence database (BIGSdb, published in 2010), enables PubMLST to include all levels of sequence data, from single gene sequences up to and including complete, finished genomes.  Here we describe developments in the BIGSdb software made from publication to June 2018 and show how the platform realises microbial population genomics for a wide range of applications.  The system is based on the gene-by-gene analysis of microbial genomes, with each deposited sequence annotated and curated to identify the genes present and systematically catalogue their variation.  Originally intended as a means of characterising isolates with typing schemes, the synthesis of sequences and records of genetic variation with provenance and phenotype data permits highly scalable (whole genome sequence data for tens of thousands of isolates) means of addressing a wide range of functional questions, including: the prediction of antimicrobial resistance; likely cross-reactivity with vaccine antigens; and the functional activities of different variants that lead to key phenotypes.  There are no limitations to the number of sequences, genetic loci, allelic variants or schemes (combinations of loci) that can be included, enabling each database to represent an expanding catalogue of the genetic variation of the population in question.  In addition to providing web-accessible analyses and links to third-party analysis and visualisation tools, the BIGSdb software includes a RESTful application programming interface (API) that enables access to all the underlying data for third-party applications and data analysis pipelines.

## Introduction

Our ability to study complex phenotypes, i.e. those that depend on the interactions of multiple components of an organism and its environment, have been enhanced during the past 20 years by the very large increases in our capacity to collect and analyse biological information. Amongst the most important of these developments have been very high-throughput sequencing methods and the informatics approaches required to interpret the large volumes of data that they generate; however, at the time of writing, there remain major challenges in realising the potential of the opportunities presented by such developments ^1^. Specifically, these data must be stored, organised, curated, interpreted, analysed, and disseminated in a usable way. Each of these steps requires a sustainable infrastructure and need to be achieved in line with appropriate standards of accuracy and openness ^2^, while meeting ethical and legislative requirements of confidentiality and data ownership ^3, 4^. The PubMLST.org databases ^5^, which are powered by the Bacterial Isolate Genome Sequence Database (BIGSdb) software ^6^, represent an approach to meeting these goals for the analysis of microorganisms, especially bacterial pathogens.

**Figure 1.**
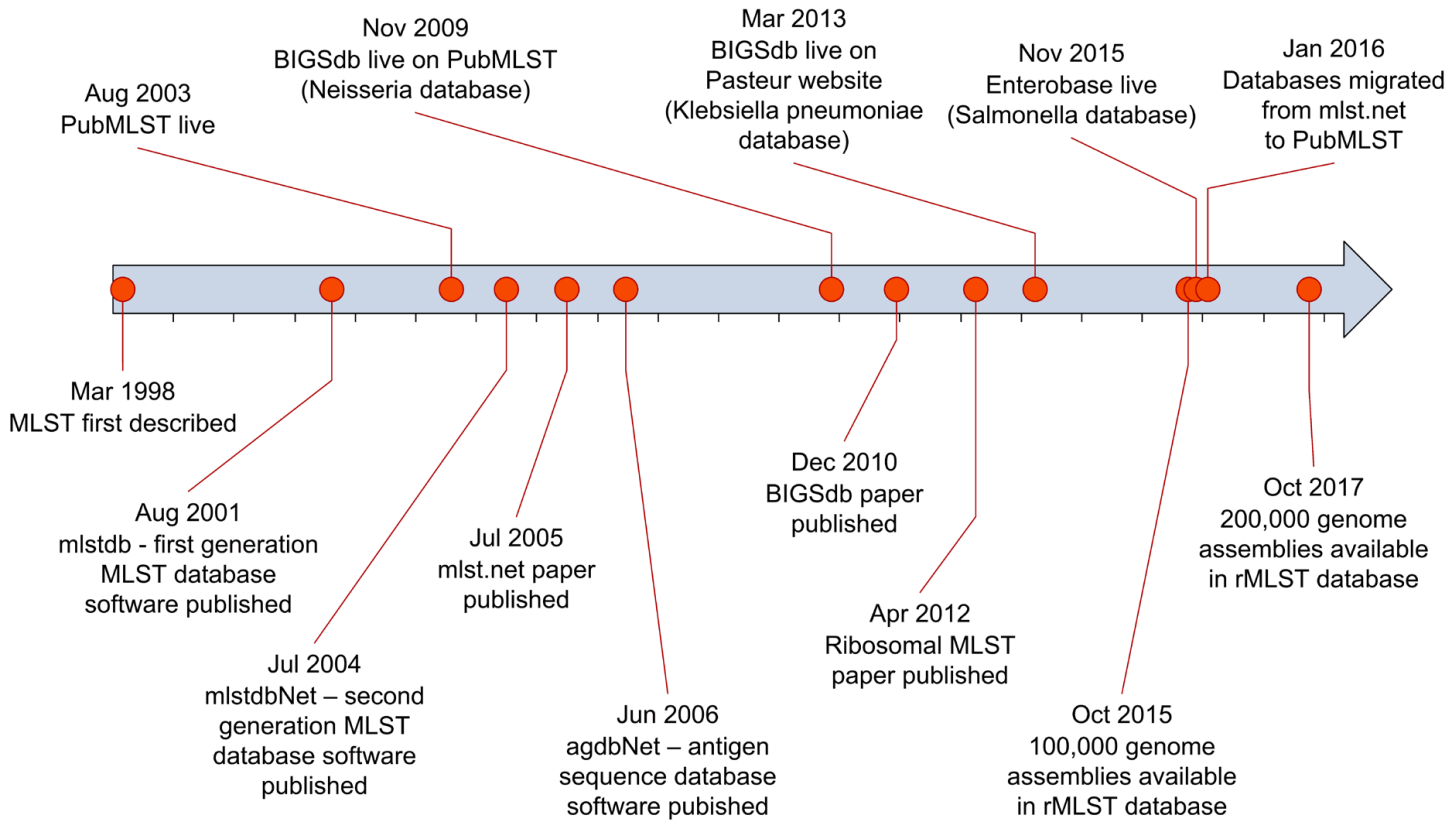
MLST comes of age: 21 years of population genomics. The PubMLST website ( https://pubmlst.org) has been running for 15 years, having been established under the pubmlst.org domain in 2003. Its immediate progenitor was the original MLST database set up to support the *Neisseria* scheme ^12, 13^, the first MLST scheme developed in 1998. The initial role of the site was to host the nomenclature and isolate collection records for typing schemes, but it was rapidly opened to the wider community, hosting schemes of other organisms ^14^. Shortly afterwards, other sites began hosting MLST schemes, the most prominent of which was mlst.net ^43^, at Imperial College, along with others at the University of Cork, Ireland later migrated to University of Warwick and subsumed within the Enterobase platform ^44^, and the Pasteur Institute, Paris, France. Early generations of software developed to support the databases ^13– 15^ were limited to specific loci defined for a single typing scheme specified in their configuration. With extensive WGS data in prospect, in 2008 work started on a platform designed to flexibly handle genomic data utilizing any number of loci and typing schemes. The resulting Bacterial Isolate Genome Sequence Database (BIGSdb) platform ^6^ has been used since then to host databases on PubMLST as well as being used for the databases hosted at the Pasteur Institute. It has been under constant development since. In 2016, the databases hosted on mlst.net were migrated to PubMLST, with the result that most MLST schemes are now hosted using the same platform (the major exceptions being *Salmonella* and *Escherichia coli*, hosted on Enterobase, although these schemes are mirrored on PubMLST).

The PubMLST.org databases employ a bacterial population genomics approach to this problem ^7, 8^. Population genomics combines the concepts of population genetics with genome-wide sequence data, to infer the links between phenotype and genotype synthesising evolutionary and functional analyses ^9^. This powerful paradigm requires an ability to link population-wide information on genome sequence data with information on the provenance (time and place) and phenotype (behaviour) of the organism in question. It is especially suited to resolving complex phenotypes ^10^, such as virulence and antibiotic resistance in bacterial pathogens, as many of these cannot be fully elucidated by *in vitro* reductionist investigations that rely on single laboratory organisms ^11^. One of the first practical implementations of bacterial population genomics was multi-locus sequence typing (MLST). MLST indexed the sequences of multiple, but few (six or seven), housekeeping gene fragments to identify bacterial genotypes and associate them with biological properties, for example the propensity to cause invasive disease ^12– 14^. The approach was later complemented by the analysis of genes encoding particular functions, such as vaccine antigens ^15^, and was ultimately expanded to enable the inclusion of whole genome sequence (WGS) data by the development of the BIGSdb platform in 2010 ^6^ ( Figure 1). The gene-by-gene approach exemplified by MLST is inherently scalable with respect to the number of loci and individual organisms included ^16^ and the BIGSdb platform has been continually developed and extended to provide additional functionality.

**Figure 2.**
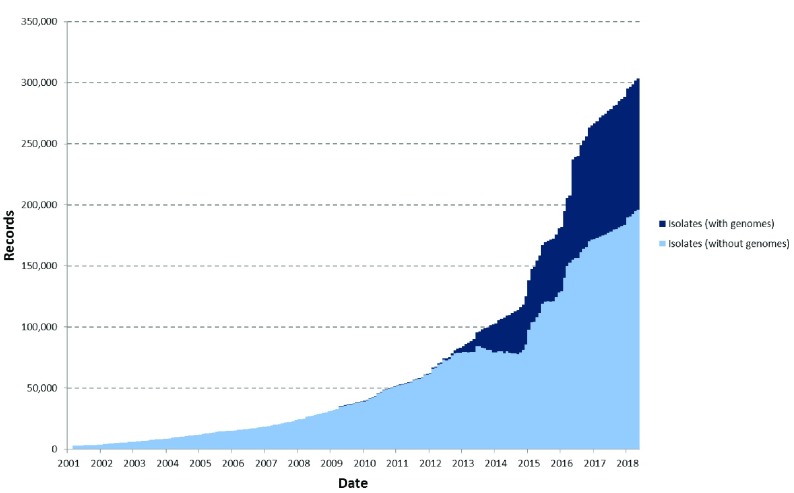
Submission of isolate records and genomes to the PubMLST species/genus-specific databases over time. Prior to 2012, most submissions consisted of provenance metadata along with MLST results and a few antigen sequence designations. Since then, the proportion of submissions that include whole genome assemblies has continually increased. The apparent dip in isolates without genomes which occurred around 2014 was due to genome assemblies being added to existing records that had been submitted previously with just MLST results.

**Figure 3.**
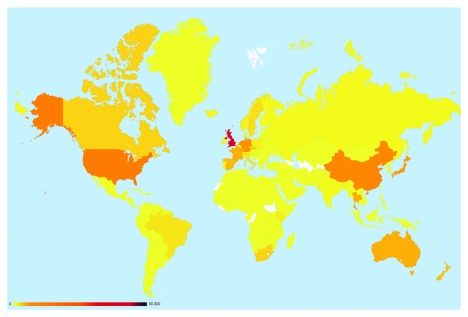
Global submissions to the PubMLST databases. Isolate records submitted to individual databases represent almost every country in the world. There are approximately 125 curators handling submissions from over 2000 active data submitters across all the species- and genus-specific databases hosted on PubMLST.

The volume of sequence data stored in the PubMLST.org databases has increased greatly with the advent of affordable WGS determination employing ‘next generation sequencing’ (NGS) platforms ( Figure 2) ^17^. At the time of writing, PubMLST hosted databases for over 100 species or genera, mainly bacteria, but also included some schemes for eukaryotes and plasmids. In principle, the BIGSdb platform can be used for any organism or virus. Many of these databases contain whole genome sequences linked to standard (seven locus) or higher resolution multilocus typing schemes such as ribosomal MLST (rMLST) ^18^ or core genome MLST (cgMLST) ^16^ ( Table 1). In total, there were more than 300,000 submitted isolate records and 100,000 genome assemblies in these databases ( Figure 2), with approximately 125 curators and 2000 active data submitters with submissions from across the world ( Figure 3). The site also hosts the rMLST databases ^18^, providing species identification and analysis tools, with approximately 15,000 unique visitors and over a million page views a month. The BIGSdb platform has been cited 990 times (August 2018, source Google Scholar).

**Table 1.** Curated data available in the ten largest databases within PubMLST (accessed July 2018; ordered by number of genomes).

Database	Isolates	Genomes	Loci	Alleles	Submitters
rMLST	223,292	223,292	53	1,350,997	-
*Staphylococcus aureus*	33,670	25,762	2,215	593,996	547
*Campylobacter jejuni/coli*	69,416	25,448	1,996	880,953	278
*Neisseria spp.*	46,985	16,511	2,979	1,170,477	343
*Streptococcus pneumoniae*	40,252	9,010	7	4,498	347
*Streptococcus agalactiae*	4,228	2,844	2,072	115,266	66
*Pseudomonas aeruginosa*	6,528	2,382	186	2,598	169
*Bordetella spp.*	1,625	1,112	1,460	35,885	12
*Acinetobacter baumannii*	3,903	936	16	2,527	166
*Burkholderia cepacia* *complex*	2,791	651	7	4,443	61

**Figure 4.**
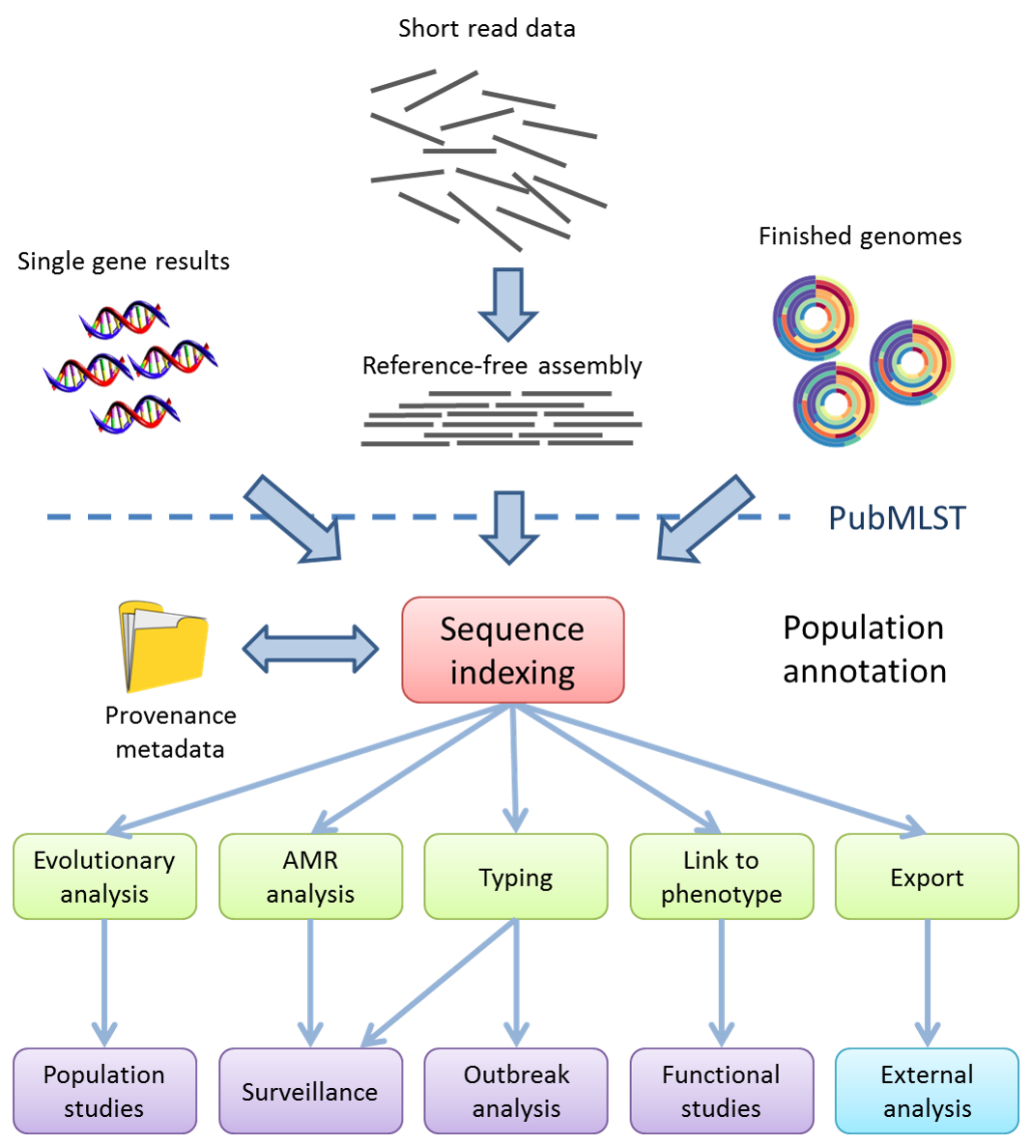
Analysis pipeline for short read data. PubMLST and BIGSdb, its underlying genomics platform, links provenance metadata with allelic sequence variation found in corresponding whole genome assemblies or sequences derived from Sanger sequencing reactions. Population annotation, the process of assigning precise variant information for loci across the genomes of large numbers of bacterial isolates, creates a structured dataset that can be used to address a range of biological questions beyond epidemiology.

To facilitate the indexing of variation across thousands of loci within each deposited genome sequence ( Figure 4) ^19– 26^, BIGSdb consists of two distinct database structures: (i) an isolate (or ‘specimen’) database that hosts provenance and genome sequence information for each sample; and, (ii) a sequence definition database that contains allelic identifiers and profiles, which provides annotation and a genetic nomenclature ^6^. Unified, curated nomenclature is essential for studies that involve very large numbers of specimens and multiple genetic loci. This separation of roles was an early design decision, reflecting the gene and allele-based paradigm used and facilitates the use of BIGSdb as a nomenclature server. Consequently, it is possible to have individual BIGSdb isolate databases acting as clients to multiple sequence definition databases and these may, in turn, each serve multiple isolate databases, creating a federated network of interconnected data resources. 

Each database in the system can have different access restrictions, with different users having roles and access rights defined by a fine-grained permissions system. This enables migration between pre-publication analyses and published data. Pre-publication data can remain private to the owner until publication but then made open-access, whilst retaining the same database identifier. As the private and the open-access data reside in the same database, it is straightforward for pre-publication or confidential data to be analysed in the context of published data. This is especially important where it may not be possible to make some or all of the data public for regulatory, legal, or public policy reasons, for example during the live investigation of disease outbreaks. All information stored within the databases is accessible to third-party applications via a RESTful application programming interface (API), depending on access restrictions ^27^. 

The hosting of typing and nomenclature information remains a central role of the PubMLST.org databases ^28– 37^ and the principle curated schemes available include: (i) conventional seven-locus MLST schemes ^12^, implemented in all databases; (ii) cgMLST schemes, for example those available for *Neisseria meningitidis* ^38^, *Campylobacter coli* and *Campylobacter jejuni* ^39^; and (iii) rMLST ^18^, available for over 223,000 bacterial specimens spanning more than 8500 species. In addition, a number of schemes for particular vaccine formulations, for example the meningococcal Bexsero Antigen Sequence Type (BAST) ^40^ or antibiotic resistance ^19, 41, 42^ are available. Although whole genome sequence data are increasingly used in the resolution of outbreaks, nomenclatures based on a subset of loci remain crucial for the development of stable hierarchical typing and nomenclature schemes used to categorise genotypes and compare them globally ^16, 45^.

## Methods

### Implementation

PubMLST functionality is provided by the BIGSdb web application, which is written in object-oriented Perl and Javascript utilizing a PostgreSQL backend ^6, 46^. It runs under the Apache web server software on Linux. The API is written as a Perl Dancer2 application interfacing with the same program libraries as the web application, run using the Starman high-performance PSGI/Plack web server ^27^.

### Operation

The PubMLST website can be accessed from any platform using a modern web browser supporting Javascript. The underlying BIGSdb software can be locally installed on a Linux machine with as little as 4GB RAM and a single processor, but at least 4 processor cores and 16 GB RAM are recommended.

***Data access*.** Both manual and automated access to data are available. Most end-users interact with PubMLST using the web interface ( https://pubmlst.org), through which all functionality is available. Querying of sequences can be performed by either pasting in data or uploading files for analysis via web forms. Complex queries can be constructed by combining search elements on a user-modifiable form and results further analysed using integrated plugins. Context-sensitive help is available within the interface linking to specific sections within an online users’ guide ( http://bigsdb.readthedocs.io). All data hosted within PubMLST is also accessible via the API, which enables machine-to-machine direct data exchange without user input ^27^. Most methods are discoverable from the root entry point ( http://rest.pubmlst.org/) and are fully documented ( http://bigsdb.readthedocs.io/en/latest/rest.html). The API is frequently used to synchronize allele and profile definitions (nomenclature), but it also supports authenticated data submissions to curator queues, isolate dataset querying and extraction of typing designations from uploaded genome sequences. This latter functionality facilitates incorporating PubMLST allele calling or use of species identification functionality from the rMLST database, directly in to an analysis pipeline ( Supplementary File 1).

***Annotation*.** BIGSdb works exclusively with assembled nucleotide sequences, although these need not be single contiguous sequences and can range from single gene sequences through MLST datasets and draft genome assemblies to complete, finished genomes present as single sequences. Therefore, genomic data present in the sequence read archives must be assembled before deposition into the database. Once these sequences have been deposited, the identification of genes at specific locations (loci) within the deposited sequence data and their precise allelic variant, is central to the BIGSdb approach to annotation. This process is referred to as allele calling and is divided into three stages.

The first stage is the identification and cataloguing of the loci (i.e *.* genes) present within the group of organisms that are being analysed in a given database. A variety of means of identifying genes within assembled bacterial genomes are available, such as PROKKA ^47^. Many bacterial species now have an annotated reference genome ^48^ and this can be used to seed the database with genes known to be present in that species. As the majority of bacteria have ‘open genomes’, a complete catalogue of the ‘pan genome’, that is all genes that are available to that group of bacteria ^49^, requires a continual process of annotation of novel genomes as they are deposited, with an ever-expanding catalogue of genes known to be present in that group of organisms. To this end, BIGSdb has no limit on the number of loci that can be stored in a database other than the storage capacity of the host computer. BIGSdb indexes each gene, which is normally synonymous with one locus in a bacterial genome, with a unique identifier that can be associated with any number of names that have been previously used. This is essential as individual annotations in bacteria from the same or related species often have incompatible names. For example, in the PubMLST *Neisseria* database a NEIS number is assigned in order of discovery to each unique gene in the database, which is linked to other names that have been used. As novel genes are identified by the on-going process of population annotation, new NEIS numbers are defined. In this way, the pan genome of the organisms in question (in this case the genus *Neisseria* as the PubMLST *Neisseria* database is genome wide) is catalogued, and a universal gene and locus nomenclature established and maintained.

The second stage is identifying the presence and location of the known genes within deposited genome data (locus identification). BIGSdb employs different strategies for this, depending on the number of loci being annotated at a given time. The most straightforward method performs a BLAST ^50^ query of the genome sequence against a database of all known alleles for each gene in turn. This is satisfactory if the results for only a few genes are being indexed, but if analysing more loci, for example for a pan-genome or cgMLST analysis where perhaps 1500 or more loci are being catalogued simultaneously, this is a bottleneck that is increasingly time-consuming as more alleles are defined. Therefore, for routine calling, the process is facilitated by reducing the BLAST search space using ‘exemplar alleles’. These are defined such that every known allele for a given gene is within 10% sequence identity of an exemplar allele of the same length. The BLAST query is then performed against a database of exemplar alleles for all loci together, which identifies the location of the loci and allows the individual locus sequences to be extracted and their allelic identities to be determined by a database lookup. This method is much more efficient and allows PubMLST to scan more than 1000 bacterial genomes for a 2000 loci cgMLST scheme within one hour on a single server using 32 cores.

The third stage of population annotation is identifying and defining the alleles present at each locus. Most of this process is automated but it requires curator oversight if a new sequence is too different from an existing allele. Again, two different strategies are employed: (i) using all existing alleles in the search; or (ii) using exemplar or type alleles to constrain the search space and prevent definition of new alleles that are sufficiently divergent to the original type allele to warrant the definition of a new gene. The first strategy is more sensitive for use when developing schemes and for research purposes whereas the second is likely to be employed routinely for schemes used for surveillance or public health purposes. For cgMLST or pan-genome automated allele assignment, new allele sequences must be within 98% identity and 98% total length of a known allele, contain terminal in-frame start and stop codons and have no internal stop codons. With type alleles employed, the allowed percentage identity difference will be increased.

As each database comprises multiple genetic loci, the BIGSdb software has the capacity to organise these loci into any number of ‘schemes’. A scheme is a group of genes (loci) that are grouped together for a particular purpose. Any locus can, in principle, be included in any scheme and there can be any number of schemes of any size. Schemes can be: (i) purely ‘typological’, for example conventional seven-locus MLST schemes ^51^, or (ii) directed to a particular biological or biochemical function, for example the Entner-Doudoroff pathway scheme developed for *Campylobacter* species ^52^; or combine both functional and typing roles, such as the ribosomal MLST (rMLST) scheme ^18^. Schemes enable hierarchical and functional analyses with easy access to defined genetic variation, providing a facile way of extracting specific sequence diversity information from large datasets rapidly and conveniently.

***Core genome MLST profile definitions and clustering*.** The bacterial core genome was originally conceived as those functional genes present in every member of a given group of related microorganisms ^53^; however, for practical purposes it is desirable to use a more relaxed definition. This is because ‘essential’ core genes may be absent from a given WGS data set for several reasons including: (i) the organism from which it is generated is a rare mutant lacking a gene normally present in all members of the species; and (ii) technical issues due to incomplete genome assembly. Both reasons would lead to an ever-decreasing core genome, as isolates without the full complement of ‘core’ genes accumulate. Consequently, bacterial cgMLST schemes, which need to be stable if they are to form the basis of nomenclature, commonly include genes present in 95% or more of WGSs ^25, 39, 54, 55^. Most isolates analysed with a given cgMLST scheme will therefore have some missing loci. To accommodate this, cgMLST profiles are usually defined with some missing loci, i.e *.* where a locus position is marked with an ‘N’ instead of an allele number. In pairwise comparisons, this will match to any allele, effectively removing the locus from that comparison, so the number of allowed missing loci needs to be kept at a low level in order to maintain resolution. The cgMLST schemes hosted on PubMLST commonly allow profile definition with up to 50 missing loci. Loci that are frequently absent from many assemblies, possibly due to them containing regions of low complexity longer than the sequence read length, are generally removed from a scheme to minimize missing data.

Once allelic profiles have been defined from WGS data, they can be clustered to identify groups of similar isolates that are likely to share a common ancestor. BIGSdb supports clustering of cgMLST schemes using a single-linkage model with multiple defined thresholds of allelic differences. These thresholds are chosen empirically and depend on the organism and the number of loci used in the scheme, but may range from 200 or more, to identify major lineages, down to 5 or fewer, suitable for identifying point source outbreaks.

***Comparative genomics.*** The BIGSdb platform incorporates a comparative genomics plugin called Genome Comparator. This performs rapid gene-by-gene pairwise comparisons of up to 1000 genomes using either: (i) loci defined in the database, generating MLST profiles consisting of the chosen loci, which can be any defined scheme or user-selected collection of loci; or (ii) using an annotated reference genome, or simply a FASTA file of sequences, as a source of comparator sequences, producing an *ad hoc* whole genome MLST analysis. A pairwise distance matrix generated from these profiles is then used to generate a NeighborNet analysis ^56^ using the SplitsTree software package ^57^ to visualise the relationships among the isolates being analysed. This is a rapid process which is suitable for identifying clusters of related organisms, for example in a disease outbreak scenario, or to find shared genetic variants when performing functional studies. Alternatively, with the alignment option selected, aligned sequences for each locus are concatenated to produce whole genome coding sequence alignments to be used for analysis in third party applications to investigate phylogenetic relationships among more distantly related isolates ^58^. As well as using genomes already in the database, it is also possible to include uploaded private genomes for analysis in context with public datasets.

Minimum-spanning trees can be constructed from allelic profiles generated from any set of loci using a plugin that exports to an integrated GrapeTree implementation ^59^. GrapeTree was specifically designed to visualise tens of thousands of whole genome MLST (wgMLST) profiles and reconstruct relationships despite missing data. Metadata, including provenance or scheme fields (such as ST or clonal complex) can be included in the analysis and used to colour nodes, producing publication quality output.

**Figure 5.**
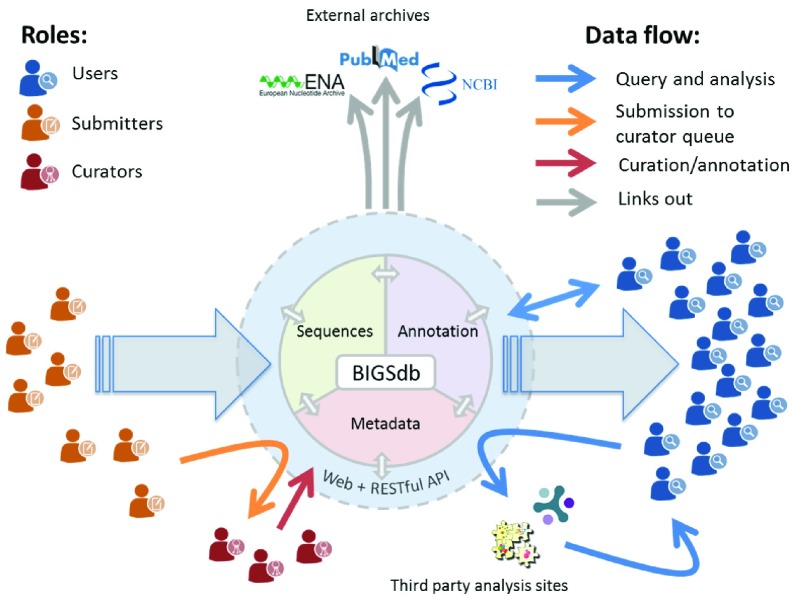
Data flow overview. Individuals may have different and overlapping roles: Users query and analyse data; submitters upload data for nomenclature assignment and inclusion in databases; and curators assign allele and profile identifiers, check metadata, upload genomes and perform allele calling (largely automated with manual oversight). Interaction with the PubMLST BIGSdb databases is via the web interface or RESTful API. Analysis of datasets returned by a query can be performed using integrated tools or forwarded to third party sites using their APIs to upload results in their required formats.

***Links to third-party analysis tools*.** PubMLST provides rich, structured datasets that can be combined with other data. Other web services provide visualisation tools and have implemented APIs so that data can be programmatically uploaded to them and various BIGSdb plugins are now available that can select datasets, link to appropriate metadata fields, and send them to these sites for analysis ( Figure 5). As an alternative to the integrated GrapeTree implementation, minimum spanning trees can be analysed using a plugin that uploads to PHYLOViZ Online ^60^. Phylogenetic trees can be generated from concatenated aligned sequences from any selected loci or set of loci defined by a scheme, and visualised with metadata overlays in Interactive Tree of Life ^61^ or combined with geographical and temporal data for visualisation in the Microreact software ^62^.

## Use cases

### Case 1: Extracting typing information from a local genome file

**Figure 6.**
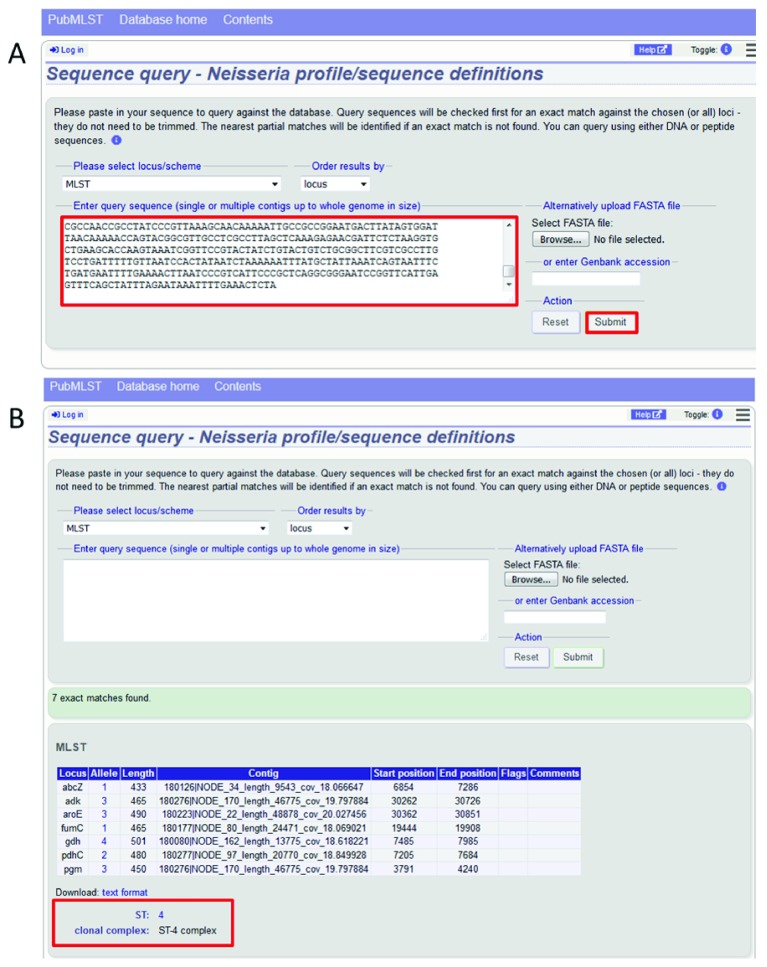
Extracting typing information from a local genome file. Typing information can be readily extracted from whole genome sequence assemblies using the sequence query page. ( **A**) Genome assembly contigs are either pasted in to the sequence query form and the required scheme or locus (in this case, MLST) selected. ( **B**) Any locus exact matches are displayed and, if this corresponds to a defined combination of alleles, the profile definition (ST/clonal complex for MLST) is displayed.

PubMLST has been used to identify sequence variants for molecular typing since its inception. When most sequencing was still performed on individual genes by Sanger methods, it was necessary to assemble forward and reverse trace files and then compare each locus assembly against known variants. This can still be done on a per-locus basis and if an exact match is not found then the most similar allele is identified along with a list of nucleotide differences, which can be manually checked to see whether the allele is novel. With whole genome data, the rapid identification and classification of microbes is considerably more streamlined. A locally assembled genome sequence can be queried against all loci of interest rapidly ^8^. This can be performed via the web interface by either pasting assembled contigs in to a search box, or by choosing to upload a FASTA file containing the contigs ( Figure 6A). Any defined scheme, such as MLST, can be selected from a drop-down box, and the analysis run by clicking the submit button ( Figure 6B). The analysis usually takes about a second to perform. In the case of MLST, individual allelic matches will be identified along with the ST if the combination of alleles has been previously defined. Alternatively, this analysis can be performed via the API allowing it to be incorporated directly in to a local analysis pipeline ( Supplementary File 1).

### Case 2: Investigating population structure

**Figure 7.**
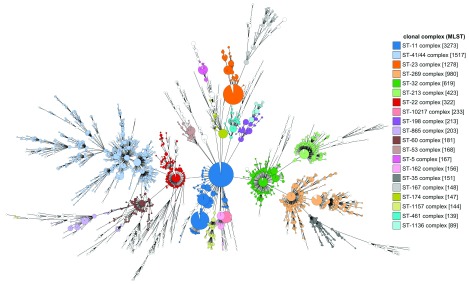
GrapeTree minimum-spanning tree of *Neisseria meningitidis* genomes (n=12,179) differentiated by cgMLST (1605 loci). A minimum-spanning tree based on allelic profiles can be generated using isolate records returned from any query and any selected scheme or group of loci. The dataset was selected by searching for species ‘ *Neisseria meningitidis*’ with an attached sequence bin size of >2 Mbp, indicative of a complete genome. Nodes are coloured by clonal complex as defined by classical MLST (7 locus), indicating strong concordance between typing schemes. Branches shorter than 150 loci are collapsed.

Bacterial populations often comprise distinct lineages of related genotypes that exhibit specific phenotypic characteristics that persist over time. These are shaped by mutation, horizontal genetic transfer and selection from interactions with, for example, the host immune system. Understanding the population structure is therefore important for epidemiology and public health intervention as well as for addressing more fundamental questions concerning evolution, persistence, and adaptation. The population structure of a microorganism can be represented using a cgMLST scheme and the integrated GrapeTree plugin to generate a minimum-spanning tree that can be overlaid with any metadata stored in the database. This provides an intuitive means to investigate and present the clustering of genotypes and how phenotypic or provenance characteristics map to these clusters. For example, performing a query in the *Neisseria* database for any isolate record where the species is ‘ *Neisseria meningitidis*’ with a sequence bin containing >2 Mbp data (indicating a complete genome sequence), identifies just over 12,000 records. By selecting the GrapeTree plugin, and analysis by cgMLST, an interactive minimum-spanning tree can be produced with nodes coloured by user-selectable criteria such as country, year of isolation or defined clonal complex designation ( Figure 7). Since the tree can be built using any combination of loci or schemes, and annotated with any set of metadata or field values determined by scheme (combinations of loci), datasets can be readily explored and relationships between genotype and phenotype identified. Analysis of a dataset of this size takes approximately 30 minutes to perform.

### Case 3: Surveillance of vaccine coverage over time

Many vaccines target expressed proteins that exhibit natural sequence variation within the bacterial population as a result of interactions with the host immune system. Over time, this may result in vaccines becoming less effective as immune pressure selects against strains with cross-reactive antigens and new lineages or variants take their place. It is therefore important that ongoing surveillance is performed to detect such changes in the bacterial population. The BIGSdb platform is flexible in how loci are defined and it allows small antigenic peptide sequences, such as those expressed on surface-exposed loops of proteins, to be used in addition to the more commonly used nucleotide sequences of complete genes. Schemes can, therefore, be defined that include only the antigen sequences of the protein components of a vaccine formulation, and variants of these defined as for any other sequence. One such scheme is the meningococcal Bexsero Antigen Sequence Type (BAST) scheme ^40^ that is being used to survey structured datasets, including carriage studies, in order to provide early warning of changes in the population of meningococci that might result in reduced vaccine efficacy ^63^.

### Case 4: Spatio-phylogenetic analysis

**Figure 8.**
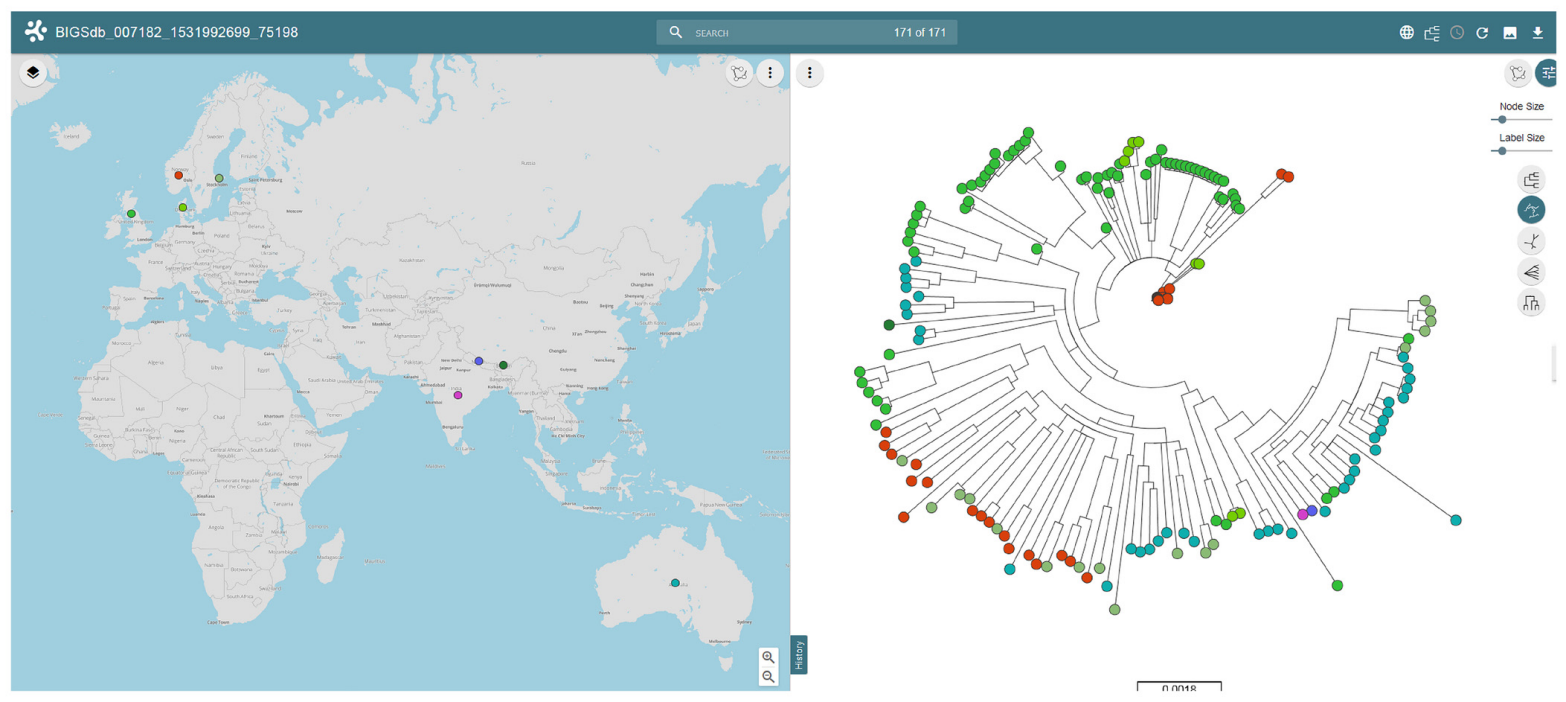
Spatio-phylogenetic analysis of the global *Dichelobacter nodosus* population. The geographical distribution of clades of *Dichelobacter nodosus* was demonstrated by analysing all genomes in the database (n=171) using the BIGSdb Microreact plugin with cgMLST loci. This created a concatenated alignment of core genes which was used to generate a Neighbor-joining tree that was automatically uploaded to the Microreact website with accompanying metadata for visualization.

The ability to plot the provenance of an isolate on a geographical map and relate this to its genotypic placement in a phylogenetic tree can be useful when investigating structuring of outbreaks or the global spread of clones. Microreact is a web tool developed to produce these visualisations and the site provides a means for data to be automatically uploaded from other resources, such as PubMLST ^62^. This can be demonstrated using data from a recent paper describing the development of a MLST scheme for *Dichelobacter nodosus*, the causative agent of ovine footrot, that indicated that the global bacterial population was geographically structured ^36^. All isolates that included a genome record in the *D. nodosus* PubMLST database (n=171) were selected and the Microreact analysis run using cgMLST loci. This generated a concatenated genome-wide alignment from which a Neighbor-joining tree was reconstructed automatically and uploaded to the Microreact web site ^62^ along with associated metadata. The geographical structuring of clades can be seen in the analysis ( Figure 8).

## Discussion

Population-scale WGS data for bacterial genomes has a wide range of applications, but is most powerful when combined with other data ^64^. Whilst providing cost-effective access to very high volumes of sequence data ^65^, the explosion of bacterial genome data generated by NGS technologies has presented challenges, first in the volume of data generated ^17^ and second in the nature of those data, in that they usually result in incomplete ‘high-quality draft’ genome sequences ^66^. The gene-by-gene approach enshrined in the PubMLST/BIGSdb platform enables a hierarchical, question-driven approach to the analysis of WGS data, founded on population genomic principles ^16^. This has the additional advantage that it is backwards and forwards compatible with single or other multiple locus analyses and isolate characterisation schemes. In addition, isolates with partial sequence data (e.g. those that have been characterised with conventional MLST) are easily included by choosing the appropriate analysis scheme for the data available ^5^. The platform has proved to be highly popular, with a multitude of schemes published on the website. Schemes can be readily established and maintained via the web interface by expert curators without the requirement of extensive bioinformatics expertise.

A vital, if apparently mundane, application is the provision of harmonised nomenclatures by means of the nomenclature server function. The BIGSdb software has been built from its inception to enable the cross-referencing of multiple nomenclatures, isolate names, and gene and allele descriptors and this has proved to be of increasing importance. As each WGS dataset, gene, isolate and allelic variant has a unique identifier within a given BIGSdb database, the PubMLST.org website can be used as a single point of reference to integrate and link information such as sequence read archive reference, GenBank accession number, PubMed id, different isolate names and designations, and different gene and allele identifiers that have been assigned by distinct annotation exercises. As this is a cross-referencing system, each of these existing nomenclatures remain in place but can be readily related to each other in combined analyses. The API provides a means whereby this information can be automatically exchanged, enhancing the capacity for data harmonisation. 

**Figure 9.**
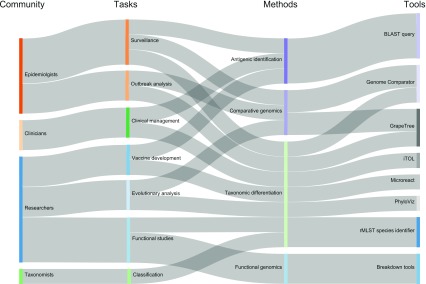
Relationships of community, tasks, methods and tools supported by PubMLST. PubMLST links structured bacterial isolate datasets with whole genome sequence data and molecular typing nomenclature to provide a rich resource that can be exploited for a wide range of tasks including surveillance, vaccine development, evolutionary analysis and functional studies.

Disease epidemiology, especially but by no means exclusively for human infections, has been and remains a major application for PubMLST.org and related databases. PubMLST is the largest host of bacterial molecular typing databases and is extensively queried and analysed by different target audiences ( Figure 9) ^40, 63, 67– 78^. Some of the larger databases now host thousands of genomes and, with the increasing implementation of cgMLST and wgMLST schemes for various organisms, offer a wide range of possibilities beyond typing and epidemiological analyses. Because the sequence variants of thousands of loci are indexed on deposition, this provides population annotation where not only the positions and identity of loci are recorded, but also their exact variant. Although the obvious utility of this is high-resolution typing, this is also the start of being able to link phenotypes to variant combinations of loci whose biochemical role has been established.

The composition of the majority of databases is contingent, as they are dependent on submission by users, who have different motivations for data submission. A principle aim of most database curator teams is to catalogue the known diversity of the target organism or group of organisms. Hence, some users submit data only to obtain new nomenclature designations; however, other users submit representative or complete sets of samples, for example those associated with a given study or publication. To accommodate this PubMLST hosts specific projects and datasets within the public databases that can be queried separately. An example of this is the longitudinal 15-year survey of the molecular epidemiology of clinical *Campylobacter* isolates in Oxfordshire from 2003–2018 ( https://pubmlst.org/campylobacter/projects/Oxfordshire_Human_Surveillance/). The 3,300 *Campylobacter* isolated from 2003–2009 were characterised with MLST with data deposited on PubMLST in near real-time ^79^, but since 2010, WGS high-quality draft sequences have been deposited ^69^. A further example is the Meningitis Research Foundation Meningococcus Genome Library, where a WGS assembly for every *Neisseria meningitidis* isolate collected from cases of invasive meningococcal disease in the UK from 2010 onwards is deposited ^7^. This has proved an invaluable resource for meningococcal research and surveillance, in particular with regard to investigations of vaccine efficacy at a time when vaccine policy with respect to this organism has been changing ^22, 40, 80, 81^.

The BIGSdb platform supports the combination of private and public data, with authorised users able to upload private data that can be shared with specified groups as required. This facilitates the use of PubMLST as a host for international multi-agency epidemiological surveillance projects, such as the European Meningococcal Epidemiology in Real Time (EMERT) database. This database was established in 2008, under the auspices of The European Meningococcal and Haemophilus Disease Society (EMGM) and allows European reference laboratories to upload partial or complete meningococcal isolate data to share among themselves, with the ability to update records as more information becomes available ( http://emgm.eu/emert/). This stand-alone system that collects serological, and MLST and antigen sequence data linked to minimal metadata, is being migrated to PubMLST shortly, allowing collection and analysis of whole genome data. Access to EMERT data will be restricted to reference laboratories and the European Centre for Disease Prevention and Control (ECDC). EMERT II will reside as a private project within the public database so that any submitted data can be analysed in the context of global datasets but these data will be made available subject to release policies, avoiding the current duplication of data submission to multiple databases. The system will integrate with the European Surveillance System (TESSy) database ^82^ *via* the BIGSdb RESTful API ^27^, facilitating automated extraction and reporting of molecular typing summaries from genome data.

PubMLST also supports international surveillance and is part of a developing ecosystem of independent third-party tools that make molecular typing nomenclatures readily available ( Table 2) ^83– 88^. The emergence of cgMLST as a method of choice for long-term and international epidemiology by a number of international agencies ^89, 90^ means that this role continues to be essential. PubMLST is well positioned to continue serving nomenclatures for this effort, along with extensive collections of structured isolate record data for a wide range of pathogenic and other bacterial species. These structured datasets coupled with extensive genomic data, complex query tools and analysis methods provide a platform for investigating a wide range of biological questions.

**Table 2.** Web services and software tools that directly utilize data hosted by PubMLST.

Service/software (ref.)	Tool type	URL
MLST-CGE ^85^	Web service	https://cge.cbs.dtu.dk/services/
GoSeqIt	Web service	https://www.goseqit.com/
Enterobase ^44^	Web service	https://enterobase.warwick.ac.uk/
MLSTcheck ^86^	Open source	https://github.com/sanger-pathogens/mlst_check
mlst	Open source	https://github.com/tseemann/mlst
SRST2 ^88^	Open source	https://github.com/katholt/srst2
stringMLST ^87^	Open source	https://github.com/jordanlab/stringMLST
MOST ^84^	Open source	https://github.com/phe-bioinformatics/MOST
MLSTar ^83^	Open source	https://github.com/iferres/MLSTar
Krocus	Open source	https://github.com/andrewjpage/krocus
Bionumerics	Commercial	http://www.applied-maths.com/bionumerics
SeqSphere+	Commercial	https://www.ridom.de/seqsphere/
CLC	Commercial	https://www.qiagenbioinformatics.com/
Smartgene	Commercial	https://www.smartgene.com/

## Conclusions

The PubMLST databases and BIGSdb software originated as part of the development of the MLST approach to the characterisation of bacterial strains in 1998 ^12^. Over the succeeding twenty years, the capacity to generate data and to interpret it greatly increased, but the fundamental requirements have remained the same, with open-access, curated, and interpreted data at the heart of the endeavour ^16^. The population genomic framework, with its foundation in evolutionary biology ^9^, provided an effective and powerful intellectual foundation for the structuring of the databases. Importantly, the approach proved to be highly scalable, enabling a transition from twelve genes in just over 100 isolates in the first MLST paper ^12^ to hundreds of thousands of isolates, characterised at thousands of loci ^44^. Despite these advances, however, the power of this approach is yet to be fully realised. The very rich data available at the time of writing has only been partially explored, especially with respect to population annotation, the cataloguing of variation across the genome with reference to biological function. While advances in analysis approaches and data integration, especially those in artificial intelligence and machine learning techniques ^91^, are likely to substantially aid the realisation of the potential of these data, we contend that engagement of individuals expert in particular organisms or systems will remain the most important contribution to the exploration of these datasets, as will the continued integration of diverse data sources by automated means.

## Data availability

All data underlying the results are available as part of the article and no additional source data are required.

## Software availability

The source code for BIGSdb, including the RESTful API application, is available from: https://github.com/kjolley/BIGSdb.

Archived source code at time of publication: https://doi.org/10.5281/zenodo.1420943 ^46^.

License: GNU General Public License v3.0.

## Supplementary material

**Supplementary File 1. Using the API to query PubMLST databases with a local genome assembly file.**

Examples are provided to demonstrate how to query a local contigs file against an MLST scheme hosted on PubMLST and how to interact with the rMLST species identification tool via the command line.
