# Supplementary material for: Open-access bacterial population genomics: BIGSdb software, the PubMLST.org website and their applications
Source: Wellcome Open Res. 2018 Sep 24;3:124. [Version 1] doi: 10.12688/wellcomeopenres.14826.1 (PMC6192448; doi:10.12688/wellcomeopenres.14826.1)
Supplement: Supplementary file 1 [file wellcomeopenres-3-16155-s0000.tgz › 70e2acdf-a11d-4f76-8053-afa160b0270c_Supplementary_S1.docx]

**Extracting MLST data from a local genome using the API**

A genome assembly file (contigs.fasta) will be queried against the PubMLST *Neisseria* sequence definition database (pubmlst_neisseria_seqdef) for MLST (scheme 1):

**(echo -n '{"base64":true,"details":false,"sequence": "'; base64 contigs.fasta; echo '"}') | curl -s -H "Content-Type: application/json" -X POST "http://rest.pubmlst.org/db/pubmlst_neisseria_seqdef/schemes/1/sequence" -d "@-"**

The first line above encodes the JSON payload for the call and then passes this to the curl program to send to the PubMLST server. Setting the details parameter to ‘true’ would additionally return the positions and orientations of the identified loci within the uploaded contigs. The response is in JSON format:

**{**

**"exact_matches": {**

**"abcZ": [**

**{**

**"allele_id": "1",**

**"href": "http://rest.pubmlst.org/db/pubmlst_neisseria_seqdef/loci/abcZ/alleles/1"**

**}**

**],**

**"adk": [**

**{**

**"allele_id": "3",**

**"href": "http://rest.pubmlst.org/db/pubmlst_neisseria_seqdef/loci/adk/alleles/3"**

**}**

**],**

**"aroE": [**

**{**

**"allele_id": "3",**

**"href": "http://rest.pubmlst.org/db/pubmlst_neisseria_seqdef/loci/aroE/alleles/3"**

**}**

**],**

**"fumC": [**

**{**

**"allele_id": "1",**

**"href": "http://rest.pubmlst.org/db/pubmlst_neisseria_seqdef/loci/fumC/alleles/1"**

**}**

**],**

**"gdh": [**

**{**

**"allele_id": "4",**

**"href": "http://rest.pubmlst.org/db/pubmlst_neisseria_seqdef/loci/gdh/alleles/4"**

**}**

**],**

**"pdhC": [**

**{**

**"allele_id": "2",**

**"href": "http://rest.pubmlst.org/db/pubmlst_neisseria_seqdef/loci/pdhC/alleles/2"**

**}**

**],**

**"pgm": [**

**{**

**"allele_id": "3",**

**"href": "http://rest.pubmlst.org/db/pubmlst_neisseria_seqdef/loci/pgm/alleles/3"**

**}**

**]**

**},**

**"fields": {**

**"ST": "4",**

**"clonal_complex": "ST-4 complex"**

**}**

**}**

**Species identification using the PubMLST rMLST database API**

A genome assembly file (contigs.fasta) will be queried against the PubMLST rMLST sequence definition database public instance (pubmlst_rmlst_seqdef_kiosk) for rMLST (scheme 1). Setting details to ‘true’ will run the taxon predictor tool.

**(echo -n '{"base64":true,"details":true,"sequence": "'; base64 contigs.fasta; echo '"}') | curl -s -H "Content-Type: application/json" -X POST "http://rest.pubmlst.org/db/pubmlst_rmlst_seqdef_kiosk/schemes/1/sequence" -d "@-"**

Response (exact matches not shown for brevity):

**{**

**"exact_matches": {**

**…**

**},**

**"fields": {**

**"genus": "Neisseria",**

**"other_designation": "ST-4 complex/subgroup IV",**

**"rST": "2504",**

**"species": "Neisseria meningitidis"**

**},**

**"taxon_prediction": [**

**{**

**"rank": "SPECIES",**

**"support": 100,**

**"taxon": "Neisseria meningitidis",**

**"taxonomy": "Proteobacteria > Betaproteobacteria > Neisseriales > Neisseriaceae > Neisseria > Neisseria meningitidis"**

**}**

**]**

**}**
