# Supplementary material for: Open-access bacterial population genomics: BIGSdb software, the PubMLST.org website and their applications
Source: Wellcome Open Res. 2018 Sep 24;3:124. [Version 1] doi: 10.12688/wellcomeopenres.14826.1 (PMC6192448; doi:10.12688/wellcomeopenres.14826.1)
Supplement: Peer review history

# Peer review: Referee response for version 1

**DOI:** 10.21956/wellcomeopenres.16155.r33973

**Published:** 2018-10-15

**Authors:** Hannes Pouseele

**Affiliations:** 1 Data Analytics, BioMérieux/Applied Maths, Sint-Martens-Latem, East Flanders, Belgium

I found the article generally well written, with a good balance between the history of gene-by-gene systems, the previous version of BIGSDb and the novelties that have been implemented lately. The use cases are clearly describing the utility of this resource and its databases for a microbiologist. The effort that was done to provide an API is a key element in allowing machine-to-machine exchange and integration in the broader digital microbiology space. As such, the article serves as a good introduction for novices and a good overview for experts of recent evolutions in the platform.

 Some more specific comments.

- On page 1, the sentence “Originally intended as a means … “ is rather long, and its meaning is not immediately clear
- On page 3, what do the authors mean by the “federated” in “a federated network of interconnected data resources”
- On page 3, the authors discuss the data access model, focusing on the private/public distinction. However, there is a third player in the game: the people that host the service itself have by definition administrator access, and maybe also curator access to all private data as well. It would be good to specify what the data access policy is for these types of access.
- On page 3, what do you mean by “the principle curated schemes”?
- On page 7, the allele detection method is described in great precision. However, the last sentence is of the first paragraph is rather vague. To what will the allowed percentage identity difference be increased? When will this be done, the formulation implies this is a future change?
- Also on page 7, it is somehow remarkable that these sequence identity related thresholds are independent of the organism. Maybe worth mentioning that these are universal thresholds, and why this can be the case?
- On page 8, the authors describe how schemes are built. It strikes me that there is no mention of filtering loci based on the presence of repeated DNA. Does this imply that there is no repeat filtering? In my experience, this is an avoidable cause of noise in the analysis.
- On page 8, first line of the second column has “colour” following British spelling. What is the journal’s policy on this? Is the spelling consistent throughout the article? I suspect the same is going on with “proved”(page 11 and 12), I would tend to use “proven” but I will leave the choice to the native speakers.

I have read this submission. I believe that I have an appropriate level of expertise to confirm that it is of an acceptable scientific standard.

# Peer review: Referee response for version 1

**DOI:** 10.21956/wellcomeopenres.16155.r33972

**Published:** 2018-10-08

**Authors:** Sylvain Brisse

**Affiliations:** 1 Biodiversity and Epidemiology of Bacterial Pathogens, Institut Pasteur, Paris, France

General comments:

 The article describes the current status of the pubMLST web site and its underlying web application software tool, BIGSdb. The article is very clearly written, concise and extremely informative as to the novel functionalities developed since the previous publication on the same subject. The developments of the platform that were achieved in recent years are quite impressive and the platform now allows any number of genomes and their linked provenance data to be analyzed at any number of gene loci for any number of microbial species/genera. Perhaps the most important developments are the APIs that allow third party software to interconnect with the database contents, effectively placing the BIGSdb nomenclatures at the center of a complex ecosystem of bioinformatics tools and resources in the fields of microbial population biology, epidemiology, clinical diagnostics and public health surveillance of pathogens.

 The impressive community of curators and users that nourish the databases and benefit from the pubMLST resource is a testimony of its unique value for basic microbiology research as well as for public health and clinical applications.

 This large success in turns raises the important issue of the sustainability of the resource. As is apparent from the authors list of the manuscript, the BIGSdb resource was almost exclusively developed by a small team of academics. The impact of this team on the field is truly remarkable, but the huge success of the pubMLST resource creates a kind of anomaly in the sense that its fragile reliance on short-term academic engagement and grant-based financial support highlights the lack of long-term, institutional committment to secure the future of the resource. The large community adoption and the dependence of increasing numbers of public health surveillance networks on this resource calls for a collective reflection on how the resource could be supported by end users or other stakeholders. An engagement by the microbiology and public health communities to support the maintenance of the database infrastructure and future developments of novel functionality seem to be a logical consequence of the large recognition of the value of the pubMLST resource, but remains to be organized. One possible way forward might be to create a more structured community of users and supporters of the resource that could promote activities such as training or community-based software development, and would foster collective actions aiming at securing long-term financial support and sustainability of the resource. In this context, the authors might want to elaborate in the manuscript, on the way communities of users are currently structured.

 Specific comments

 The pubMLST home page is concise and focused on rapid access to main functionality or information and clearly seem to be designed for people familiar with the site purposes. It might be useful to add a short header text summarizing the purpose of the web site for non familiar users, with some links to key publications like the one under consideration.

 Figure 8: the tree is derived from a contatenation of alignments of gene sequences of a given scheme.  This is a very useful BIGSdb functionality as it allows capturing the entire sequence variation rather than just its summary based on allele identifiers. However in this case, the impact of homologous recombination on inferred strain relationships is not taken into account; a recombination purging step might be a useful addition.

 page 12: The composition of the majority of databases is contingent : what is meant by “contigent” here?

 page 12: A “principle” aim of most database curator teams is to catalogue the known diversity : is “principal” meant?

 Figure 9 is a useful overview of different ways the platform can be used by different user communities. Although it is largely self-explanatory, there is no discussion about this figure in the text.

I have read this submission. I believe that I have an appropriate level of expertise to confirm that it is of an acceptable scientific standard.
